# Mr. Thomas Sandwith's Observations in Medicine and Surgery

**Published:** 1821-09-01

**Authors:** 


*Observations in Medicine and Surgery.* By THOMAS SANDWITH, Surgeon, Beverly.

(Continued from Vol. II. p. 215.)

MR. T. SANDWITH begins his Observations with *cases of protracted inflammation of the pericardium.* These are prefaced by some sensible remarks on blood-letting, which operation he thinks ought to be regulated by the nature of the part inflamed. Hence, in clearly marked cases of inflammations of the mucous membranes, he says, that it should be made a rule to bleed the patient while upright, and to make a large orifice; and, when the serous membranes are inflamed, our patients should be bled in the horizontal posture, in order that a large volume of blood may be abstracted, before a disposition to syncope is induced. The latter mode of venesection is applicable to inflammation of the vital organs, and, according to the author, also, to inflammation of the ligamentous coverings of the joints, as in acute rheumatism. Now, with respect to the last mentioned disease, there has existed much difference of opinion respecting the necessity and even propriety of phlebotomy; and we have of late been induced to believe, that acute rheumatism may generally be cured by a judicious diversion of the circulation to the skin, kidneys, or intestines. The membranes, which envelop the vital organs, are not more liable to a transition of arthrosial* inflammation, when bleeding is avoided, than when it has been employed; and we find that, after the most ample abstractions of blood, fatal migrations of inflammatory action are daily taking place; as from the pleura to the serous coat of the intestines, and from either to the membranes of the brain, &c. and from one kind of structure to another, as from the mucous to the serous surfaces.

In the inflammation of serous membranes Mr. S. prefers a small to a large orifice in the vein, and says that when a cure does not follow the first bleeding, the operation should be repeated every two, four, or six hours, until it does. The quantity of blood to be withdrawn cannot be precisely determined; and Mr. S. thinks that we have no alternative, but to go on bleeding according to the method of Valsalva, until the powers of life are too feeble to support inflammatory action. Two cases of protracted inflammation of the pericardium are brought forward to prove the utility of repeated bleedings, and Mr. S's conduct in the treatment of these cases reflects much credit on his judgment and practice. The word *essera,* which occurs in the description of one of the cases, and is more properly written *eshera,* is an obselete term introduced by Plouquet, signifying summer-rash, or a variety of lichen. The etymology of *jumentous* we are at a loss to discover, except the author derives it from *jumentum,* which is not sufficiently explicit and defined to be used as a descriptive term, applied to the urine.

2. *On the Utility of Blood-letting in bleeding Diseases.* An instance of purpura hæmorrhagica and two of menorrhagia are adduced to evince the striking advantages of venesection in these diseases, which were formerly supposed to arise from debility, and to require an opposite treatment. We met with an obstinate case of hæmorrhagic purpura in a boy, which was cured by calomel and jalap administered every second day in purging doses, and bark and sulphuric acid on the intermediate days. Had his friends consented to bleeding, we have no doubt the disease would have been sooner removed. With respect to menorrhagia, we have always found bleeding the only effectual remedy, where the pulse has been quick. The hæmorrhage in these diseases seems to be a spontaneous, but inadequate effort to terminate an over-excited condition of the vessels; and, like the

---

* From ἀρθρον, *artus,* or ἀρθροω, articulo; whence arthrosia, which signifies gout and rheumatism, in which sense Mr. Good employs it.

secretions from the serous and mucous membranes, continues until the volume of blood has been reduced, or the balance of the circulation has been restored by exciting some other evacuation.

3. *Passive Congestion of the Lungs.* "There is a disease of the chest in which the right chamber of the heart, the pulmonary artery, and the venous system of the liver, are overcharged. The blood is imperfectly arterialized, and the system is supplied by the left side of the heart imperfectly. The symptoms of this disease are a feeble pulse; a cold, pale, and relaxed skin; painful struggling in the region of the heart, which beats violently, while the pulse at the wrist is weak; shortness of breath, dyspepsia, and occasional syncope. Cordials aggravate this condition of the heart and lungs; and should not be used even when the patient is in a condition resembling syncope. It is pleasing in such circumstances to see a debilitating power, *viz.* the lancet, restore animation." P. 41.

Two short cases, but very instructive ones, follow.

4. *On Erysipelas from the Bites of Leeches.* Two cases are related, in one of which most profuse bleedings from the arm and temporal artery were necessary to subdue the delirium and other symptoms of violent excitement; and the other case, in which less vigorous measures were pursued, terminated in the death of the patient.

5. *On the Pathology of the Mucous Membrane of the Larynx, Trachea, and Bronchia.* The remarks on the inflammations of the larynx, trachea, and bronchia, are very brief, and appear to be introduced with the view of representing the utility of bleeding.

6. *Cases with Dissections.* These are few, but important. The remarks on apoplexy and a case of scirrhus in the pancreas we shall lay before our readers.

"Between inflammation of the brain and apoplexy, provided we take in the whole disease, there is no essential difference. The former seldom confines itself to that nosological description, but appears in various forms, and apoplexy is one of them. The symptoms which constitute pyrexia, have ranked phrenitis with the phlegmasiæ; and if the premonitory symptoms of apoplexy be carefully noted, its exclusion is unaccountable. The occurrence of fever after the fit has been remarked by all observers, and the other symptoms are analogous." 67.

*Scirrhous Pancreas* is a rare disease, and its early symptoms being found equivocal, it has always been considered most difficult to detect. Mr. S's case and dissection will therefore be acceptable.

"Mrs. C. an unmarried lady, sixty-seven years of age, of a dark complexion, complained of continual pain in the epigastric region, which extended to the left hypochondrium. Deep pressure increased the pain, and there was a remarkable pulsation below the cartilages of the false ribs on the left side. She was a good deal emaciated, but without fever; the bowels were generally costive. Her appetite was bad; but she had no sickness or vomiting. Her complexion was sallow, and her eyes had a peculiar expression of anxiety. The pain was sometimes most intense, and on these occasions she exhibited signs of distraction. Her body was agitated in a most extraordinary manner; she tore the bed clothes, and said she could tear the flesh from her bones. Large doses of laudanum afforded some relief. Six weeks before her death, a vomiting came on. Every thing she swallowed was rejected, and sometimes a little bile and mucus. Nothing appeared to relieve this distressing symptom, and after six weeks of extreme misery she died.

"*Dissection.* The cadaver was extremely emaciated. The spine could be distinctly traced through the parietes of the belly. The stomach was erythematous. The pancreas had the usual marks of scirrhus; and the splenic artery was imbedded in scirrhous matter. A small tubercle was found in the parietes of the uterus, and the germ of a polypus in its cavity. All the other viscera were sound." 90.

### II. SURGICAL OBSERVATIONS.

1. *On Contractions in the Neck.* Mr. Sandwith thinks there are contractions after burns, for the removal of which Mr. Earle's method of excision cannot be employed; and he introduces two cases to prove his assertion.

In the first case, the chin was found adhering to the sternum, and had been in that situation twelve months. The substance, covering the front of the throat and constituting the bond of union between the parts above-mentioned, consisted of a material, resembling gristle.. The face was greatly deformed by the accident. An incision was made through the cicatrix half an inch deep and four inches in length. At first only the space of an inch and a half was obtained; but by confining the chest and throwing the head backwards in bed, the whole lost space was restored, and the countenance resumed its original appearance. After a while a collar was put round the neck. This was made of unyielding materials, and its size increased every third or fourth day, which, by tearing open and retarding the too rapid healing of the skin, had the effect of completing the cure.

The general appearance of the second child was still more formidable than that of the first.

"The face had suffered more; the shoulders almost touched the ears; and the collar-bones were nearly dislocated; in addition to which, a contraction, like a leather thong held the fore-arm at an acute angle with the arm." 101.

"A greater number of vessels were wounded during the operation, and the external jugular veins being massed in the cicatrix, were both divided. The progress of the cure was frequently interrupted by sloughing, and the discharge was excessive. Nevertheless, after five months attention, both to the wound and the constitution of the patient, it was completed.

"Upon the arm the operation described by Mr. Earle was performed, and succeeded admirably." 102.

An operation, which reflects great credit on Mr. S. was performed also on a contracted and diseased mastoid muscle, with success. The contraction followed scarlatina. On the third day intense pain in the wound came on, and the patient was in imminent danger; the functions of the brain and head being disordered. Suppuration however coming on, and a free discharge following, every thing afterwards went on well; and by the aid of the collar, a cure was obtained.

2. *On Diseases of the Sublingual and Submaxillary Glands*. The nature and treatment of kamila is well known. The inflammation of the suppurative kind, which attacks the sublingual and submaxillary glands is always tedious, and often alarming. We have seen high fever, delirium, subsultus tendinum, and extensive sloughing of the cellular substance, and of the platysma myoides, as well as the loss of several teeth on the lower jaw, follow this disease, when a timely opening has not been made. Mr. S. most judiciously advises a depending aperture to be made under the chin with the view of draining off the pus, and of preventing it from becoming acrid and offensive, and thus obviating the disagreeable and dangerous consequences we have mentioned. Whenever purulent matter is confined by a fascia, it should always, if possible, be evacuated by an operation, both to prevent the destruction of the adjacent parts and the accession of irritative fever. In proof of the good effect of this practice, Mr. S. adduces two cases, which are highly creditable to himself and his friend Mr. Brereton.

3. *Hydatid Tumours in the Female Mammæ*. The great attention which has of late been directed to morbid anatomy, has enabled us to excel our predecessors in delicacy of discrimination, and in correctness of judgment. To Mr. Abernethy in particular we are indebted for the most scientific classification of tumours, and the most defined notions of their nature and their distinguishing features. This part of modern surgery has also been greatly enriched by the remarks of Sir Astley Cooper. Not only has the science been advanced, but the prospects of the patient improved by these refinements. How consolatory to the patient is it for a skilful surgeon to satisfy her that the ramifications of a supposed cancer are nothing more than the diverticuli or pouches of an hydatid!

"There are tumours of the female breast, which to a superficial observer present the appearance of scirrhus. These contain a fluid, are of an hydatid structure, and are distinguishable from scirrhus by striking characters. In scirrhus the skin has a dull leaden hue; in the hydatid tumour its complexion is natural. A peculiar uneasiness characterises the hardness of scirrhus, but in the other disease, the tumour is smooth, and there is no more inequality than would naturally arise from the clustering of two or more hydatids. A scirrhous tumour adheres to the skin, and gives it a puckered appearance, but in this disease the skin is smooth." 124.

The above is Mr. S's description of hydatids in the female breast; and he adduces two cases, in both which the operation succeeded. One of his patients, however, had to contend with an inflammation in the mucous membrane of the intestines.

3. *Subcutaneous Nævi.* Mr. S. cured this disease in one patient by a compress dipped in a solution of sulphate of zinc. The tumour was seated on the occiput. Two cases are related by a friend, in which the nævi were situated on the face between the eye, the cheek, and the nose. These were extirpated.

When astringent lotions and pressure fail, the knife cautiously used is the best cure. We have cut out two, from the temples, of great magnitude in very young children; and any considerable hæmorrhage was prevented by an assistant immediately compressing the mouths of the arteries with the fingers, as in the extirpation of a cancerous mamma. Care should be taken to remove the whole of the disease, and with this view the incisions should extend a sufficient distance beyond it. When the cells happen to be wounded the bleeding is profuse. Two nævi in the neck in two of our patients were cured spontaneously by suppuration; and one, which occurred to one of our own children on the side of the head, gradually subsided, and when the child was two years old was obliterated. This and those which we removed with the knife pulsated violently, but the others which got well after the suppurative process had no pulsation. Those which pulsate are probably aneurisms from anastomosis; while those which are quiescent are the aneurismal varices, and are, we think, seated in the subcutaneous veins.

4. *Case of imperfect Union of a fractured Fore-arm cured by Pressure.* This accident occurred to the author, and was cured, at the suggestion of Sir. A. Cooper, by the application of a piece of saddlers' leather, furnished with straps, in order that the pressure might be increased or diminished, as occasion required. In a few weeks, by this contrivance, the bone was perfectly united.

A few other subjects of minor importance we have omitted for want of room; but this, we trust, will be no disappointment to our readers, who we think will be desirous of seeing the book itself. We are truly happy to find the provincial surgeons display so much professional and literary excellence, as we have had occasion to discover in numerous instances; and it must be gratifying for them to reflect that every day affords them additional opportunities of acquiring the earliest information on every medical subject through the medium of periodical publications, and the liberality of those who fill the highest offices in the profession. Mr. H. and Mr. T. Sandwith have established their reputation both as practitioners and as men of science; and we doubt not they will obtain that extensive patronage and esteem, which they so eminently deserve.

---

